# Internal Tube Occlusion with An Easily Removable Non-Absorbable Double Suture: A Novel Surgical Technique Adjunct for Non-Valved Glaucoma Drainage Devices

**DOI:** 10.3390/vision7010014

**Published:** 2023-02-24

**Authors:** Alfonso Savastano, Gloria Gambini, Maria Cristina Savastano, Matteo Mario Carlà, Clara Rizzo, Tomaso Caporossi, Emanuele Crincoli, Stanislao Rizzo

**Affiliations:** 1 Ophthalmology Unit, Fondazione Policlinico Universitario A. Gemelli IRCCS, 00168 Rome, Italy; 2 Ophthalmology Unit, Catholic University “Sacro Cuore”, 00168 Rome, Italy; 3 Ophthalmology Unit, Department of Surgery, University Hospital, 56100 Pisa, Italy

**Keywords:** glaucoma, long tube, drainage device, Baerveldt, glaucoma surgery

## Abstract

To describe a surgical variant for non-valved glaucoma drainage device implants using an easily removable non-absorbable double suture into the lumen of the tube. A retrospective, non-comparative case series of 10 patients who underwent a non-valved glaucoma drainage device implant with an endoluminal double-suture for refractory glaucoma. The sutures were easily removed postoperatively without the need for an operating room. Intraocular pressure, number of medications, and early and late complications were evaluated with a follow-up of 12 months. None of the eyes that underwent an operation developed early or late complications. The first endoluminal suture was removed in all eyes with a mean time of removal of 30 ± 7 days. The second suture was removed in all eyes with a mean time of removal of 90 ± 7 days. No complications were noted, either, after or during suture removal. The mean preoperative IOP was 27.3 ± 4.0 and the postoperative IOP, at the end of the follow-up, was 12.7 ± 1.4. At the end of the follow-up, six patients (60%) achieved complete success and four patients (40%) achieved qualified success. In conclusion, in our case series, the surgical variant allowed for a safe and gradual regulation of the flow during postoperative management. Considering the efficacy of non-valved glaucoma drainage devices, an improvement in the safety profile allows surgeons to broaden the surgical indications.

## 1. Introduction

As has emerged in recent reports, the indications for glaucoma drainage devices (GDD), typically used in refractory glaucoma cases, are expanding [1,2,3,4]. All the devices have the same design consisting of a silicon tube connected to an endplate, which is placed under the tenons capsule in the equatorial region [5,6]. Valved implants have a restriction of flow that prevents hypotony (IOP less than 8 mmHg) [7]. Non-valved implants have no restriction of flow, thus, surgical precautions are needed to temporarily limit the flow before plate encapsulation, which occurs after 4–6 weeks [8,9]. Most of the concerns related to the complications in tube shunt surgery, especially non-valved tubes, are concentrated on hypotony and hypotony-related complications. Choroidal effusion, choroidal detachment, and hypotony maculopathy are serious vision-threatening complications that occur mainly in the early postoperative period. In order to prevent postoperative hypotony, several precautions must be adopted during the non-valved tube implantation [10,11,12,13,14,15]. The external tube ligation can be performed with absorbable sutures, which will dissolve in 4–6 weeks, thereby providing time for fibrous capsule formation [16,17]. The internal tube occlusion is currently performed using a non-absorbable single suture into the lumen of the tube, which modulates the flow inside the tube and it can also be removed after fibrous capsule formation. Glaucoma surgery requires intense and meticulous postoperative management, where the key element is flow modulation. The combination of both external tube ligation and internal tube occlusion allows the surgeon to have a two-step modulation of the flow in the postoperative period. Here, we describe a novel surgical technique of tube occlusion with a non-absorbable, easily-removable double suture in the lumen of the tube, which can expand the possibilities of flow modulation.

## 2. Materials and Methods

### 2.1. Study Design

The study was conducted in accordance with the tenets of the Declaration of Helsinki and informed consent for the surgical procedure was obtained from all participants. All the authors reviewed the manuscript and testify to the accuracy and completeness of the data in addition to the adherence of the study to the presented protocol. The study was designed as a single-center retrospective non-comparative case series. Participants were enrolled at the “Fondazione Policlinico A. Gemelli IRCSS” in Rome, Italy. Data, as well as all the patient investigations performed in this study, have been deposited in the REDCap system of Fondazione Policlinico Universitario A. Gemelli IRCCS Data Center, Rome, Italy. The indication for Baerveldt (BAE) (Johnson &Johnson, Santa Ana, CA, USA) implantation was the diagnosis of refractory glaucoma with uncontrolled IOP, despite medical therapy and the presence of conditions that make filtering surgery less successful, such as a history of previous glaucoma filtering surgery or drainage implant surgery, and/or the diagnosis of secondary glaucoma (uveitic glaucoma, neovascular glaucoma, or traumatic glaucoma).

The study was conducted in accordance with the Declaration of Helsinki and approved by the Ethics Committee of “Università Cattolica del Sacro Cuore”. The surgeries were performed by two expert surgeons (S.R. and A.S.) from January 2020 to February 2021.

### 2.2. Surgical Technique

**Figure 1:**
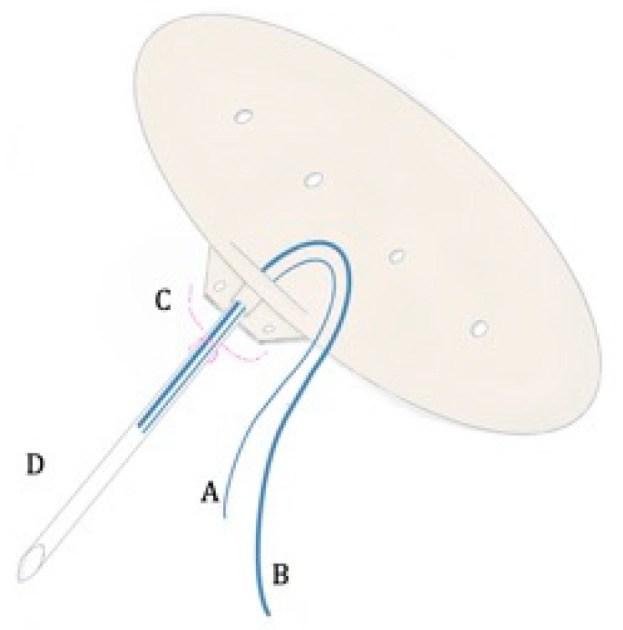
Graphic representation of the Baerveldt device. A: 6-0 Prolene suture into the lumen; B: 4-0 Prolene suture into the lumen; C: water-tight Vicryl ligation; D: area of the three fenestrations.

**Figure 2:**
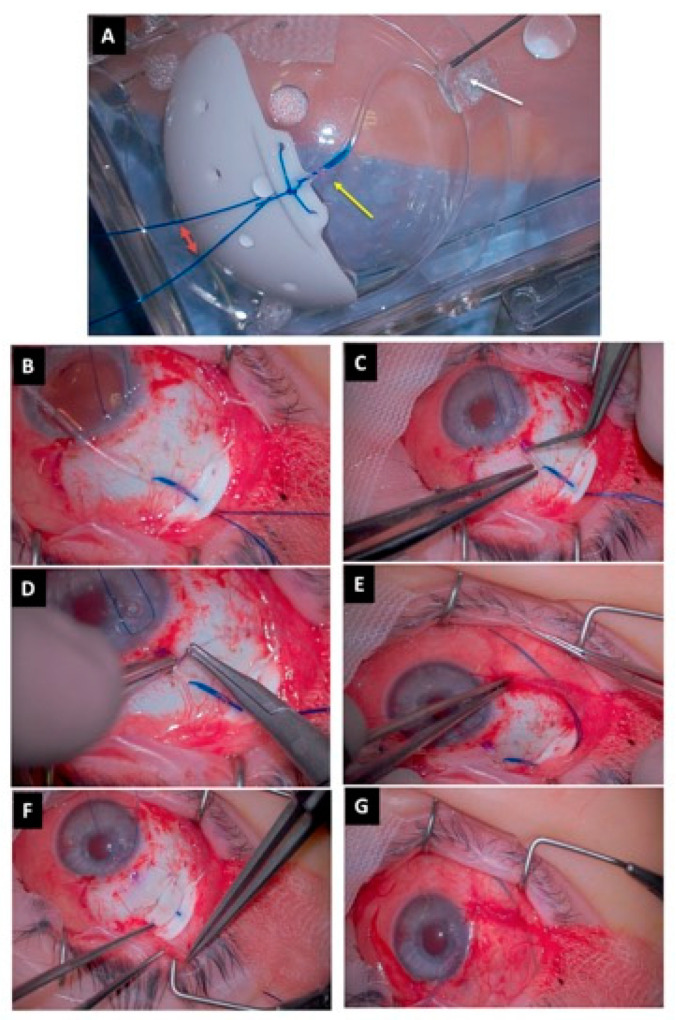
Surgical phases. (**A**): Long tube (Baerveldt) preparation. Two Prolene sutures (4-0 and 6-0, respectively) are positioned inside the tube (double-headed red arrow) and tightened through a Vicryl 7-0 (yellow arrow). The water-tight closure test is performed by injecting BSS into the tube and observing no fluid leakage on the plate of the device; (**B**): Baerveldt device positioned into the superotemporal quadrant and fixed at 10.0 mm from the limbus. The wings are positioned under the superior and lateral rectus muscles; (**C**): introduction of the tube into the anterior chamber after its trimming; (**D**): three fenestrations of the tube are performed in order to avoid postoperative IOP spikes; (**E**): both Prolene, 4-0 and 6-0, sutures are gently positioned under the conjunctiva reaching the inferotemporal quadrant; (**F**): the human scleral patch is used to cover the tube; (**G**): conjunctival suture using 7-0 Vicryl.

When using the Baerveldt device, the surgical procedure requires the isolation of the lateral and superior rectus muscles with muscle hooks, and the wings of the plate are placed under each rectus muscle. We attempted to exclusively open the conjunctiva laterally to the rectus muscles, in order to isolate them and achieve a good positioning of the wings of the plate underneath them. Since the Baerveldt is a non-valved device, maneuvers were performed to avoid postoperative hypotony. After device priming and fixation to the sclera around 10 mm from the limbus, using the proper holes in the anterior edge of the plate, two sutures were placed into the lumen (6-0 and 4-0 Prolene); then, we stitched a water-tight suture using a Vicryl 7.0 ligation near the tube–plate junction. (Figure 1 and Figure 2) Three fenestrations were performed proximal to the ligation using the needle of the Vicryl 7-0. Before positioning the tube into the anterior chamber, the 2 Prolene sutures were placed below the conjunctiva, passing anteriorly to the lateral rectus muscle, which made them extremely visible in the inferior temporal quadrant. This allowed for later extraction at the slit lamp after 1 month (6-0 Prolene) and 3 months (4-0 Prolene), respectively (Video S1). No viscoelastic was left in the anterior chamber due to the high risk of postoperative IOP spikes (due to the water-tight Vicryl ligation). In the end, the conjunctiva was sutured after securing the tube with a human sclera donor patch. Subconjunctival injections of gentamicin and dexamethasone were given at the end of the surgery (Figure 2).

### 2.3. Postoperative Management

The postoperative topical antibiotic was applied 6 times per day for 2 weeks. The postoperative topical steroid was administered 6 times per day for 2 weeks, 4 times/day for 1 month, 3 times/day for 3 months, tapering to twice daily for 2 months, and once daily for 1 month. Topical and systemic antiglaucoma therapy was provided to control the IOP, depending on the course of the single case (Table 1). The postoperative examinations were performed on day 1, week 1, week 2, month 1, month 2, month 3, month 6, and month 12. Postoperative adjustments to reduce intraluminal outflow resistance and to improve filtration were scheduled as follows:

- 4 weeks postoperatively: thinner endoluminal suture removal.
- 12 weeks postoperatively: thicker endoluminal suture removal.

### 2.4. Data Collection

The data obtained from the medical records of each patient that underwent BAE glaucoma drainage device implantation included their demographic, glaucoma diagnosis, preoperative and postoperative IOP for each follow-up, IOP before and after the thinner ripcord removal, and IOP before and after the thicker ripcord removal. Hypotony-related complications, such as choroidal effusion, shallow or flat anterior chamber, and hypotony maculopathy were recorded. The necessity for postoperative AC manipulation, such as viscoelastic injection or paracentesis, and for a second surgery was also recorded. The full ophthalmic preoperative examination was performed, including slit-lamp biomicroscopy, gonioscopy, and fundus examination (SL9900 Slit Lamp, CSO, Florence, Italy). The same examiner performed all the preoperative and postoperative measurements with a Goldmann applanation tonometry (G.G.). The IOP was measured three times at every follow-up, with a 5 min interval between measurements, and the average of the three, approximated to the entire number, was considered for every patient. Loss of IOP control during the follow-up was defined as:

- Hypotony: IOP < 5 mmHg on 2 consecutive visits.
- Hypertony: IOP > 21 mmHg on 2 consecutive visits.

Both hypotony and hypertony were defined as transient if they were present for more than 3 weeks yet less than 6 weeks. Conversely, they are defined as persistent if they occur for more than 6 weeks [18].

Complete success is defined as a final IOP of less than 21 mmHg, a reduction of 20% of the baseline IOP, absence of hypotony or complications, and without a need for reintervention. Qualified success is defined as the abovementioned characteristics reached within the adjunct of medical therapy.

## 3. Results

Ten patients (M = 5, F = 5) were retrospectively included in the case series. Table 1 shows the demographic characteristic of the patients. All patients had uncontrolled IOP despite maximum medical therapy before surgery. Six eyes had already undergone previous glaucoma surgery: trabeculectomy (*n* = 3), ExPRESS valve implantation (*n* = 2), and deep sclerectomy (*n* = 1). Two patients had neovascular glaucoma, while three patients had uveitic glaucoma.

The follow-up period was 12 months. The mean preoperative IOP was 27.3 ± 4.0 and the postoperative IOP, at the end of the follow-up, was 12.7 ± 1.4. At the end of the follow-up, six patients (60%) achieved complete success, and four patients (40%) achieved qualified success. (Table 1) No early complication that was related to hypotony emerged in our case series, such as a flat anterior chamber, a choroidal detachment, suprachoroidal hemorrhages, or severe hypotony. None of the patients required anterior chamber refilling with viscoelastic or any other intervention to solve a postoperative complication. For one patient, the removal of the suture required the operation room due to poor cooperation from the patient. In all other cases, both sutures were removed using the slit lamp. The first 4-0 Prolene suture was removed from all eyes with a mean time of removal of 30 ± 7 days. The IOP after the 6-0 Prolene suture removal decreased from 17.6 ± 2.3 to 15.1 ± 2.3. No complications were documented after the first Prolene suture was removed. The second Prolene suture was removed from all eyes with a mean time of removal of 90 ± 7 days. The IOP after the 4-0 Prolene suture removal decreased from 16.9 ± 2.4 to 11.7 ± 2.2. No complications were noted after the 4-0 Prolene suture removal. No transient or persistent hypotony emerged from our case series. Likewise, no transient or persistent hypertony emerged. Moreover, none of the cases reported any changes in their visual acuity during the follow-up period.

## 4. Discussion

There is a growing interest in tube shunt procedures. These have been mostly used over the previous decades for refractory glaucoma and complex cases; however, are now even emerging as the first choice in non-refractory glaucoma cases [1,2,3,4].

The long-term results, in terms of efficacy, of the ABC study that compared non-valved tube shunts to the valved tube shunts showed that the BAE implant is more effective in lowering the IOP than the valved one [19]. Similar results have emerged from the AVB study, in which long-term surveillance of the BAE was superior to the Ahmed group. In terms of complications in non-valved glaucoma, implants were shown to have an increased risk of hypotony when compared to valved tubes [15]. In the pooled data analysis of the ABC and AVB studies, the cumulative failure rate at 5 years was higher in the Ahmed group, and a high IOP was the main reason for the failure. However, if failure due to hypotony is considered, it occurs at a higher rate in the BAE group than in the Ahmed group [20,21].

As the efficacy of non-valved glaucoma drainage devices has been well established, the hypotony-related complications remain a concerning limitation of the technique. Hence, regulation of the flow in the early postoperative period is of remarkable interest to glaucoma surgeons and their target of surgical technique innovations, such as the one presented in this research. Currently, the management of late hypotony after tube shunt implantation includes device removal, downsizing of BAE, and tube ligation, thus, most of the precautions in regulating the flow must be taken during device implantation in order to adequately manage the flow during the postoperative period and avoid late hypotony [22,23].

Several studies have focused on the ligation of BAE tubes with an ab-externo absorbable suture that will dissolve in 5–6 weeks [7,16,17,24,25]. Even if by that time the fibrous capsule around the plate is already formed, the dissolution of the tying suture can lead to a sudden decrease in IOP with anterior chamber shallowing. Emerick et al. reported hypotony in 25% of eyes following ligature autolysis, where no occluding stent was present [26]. A second level of flow modulation was reached by placing a suture inside the lumen of the device. The endoluminal stenting is currently performed with a single non-absorbable suture of different sizes from 3-0 to 5-0 [18,27,28,29]. This will lead to better control of the IOP before and after ab-externo suture dissolution. In fact, the suture is left accessible at the slit lamp and can be removed to open the device while the ligating suture is still in place, or it can be left in place during the dissolution of the ligating suture to avoid an abrupt reduction of the IOP.

However, it has to be considered that in the early postoperative period, after tube occlusion with stenting, and after ab-external tube ligation, the IOP is expected to be high despite medication. For this reason, fenestrations are usually performed to avoid high postoperative IOPs, especially in advanced glaucoma cases. A wide variety of fenestrations techniques are described, which attempt to provide a temporary IOP-lowering effect before the release of the external ligation [17,27,30,31]. The fenestration can be created with a blade or a needle and can vary in number. There is no standardized technique, and in previous studies, a higher rate of hypotony and hypotony-related complications with spontaneous resolutions have been described in cases where fenestrations were performed [32]. Yadgarov et al. described a tube fenestration stented with a non-absorbable suture that was used to control early postoperative IOP and they reported better control of IOP compared to previous reports using tube fenestration without stenting [33]. It is well known that modulation of the flow after surgery is essential for the success of the procedure, not only for avoiding early hypotonia but also for avoiding the hypertensive phase. The hypertensive phase is defined as an IOP > 21 mmHg in the 3 months after surgery, with no evident causes [34,35,36]. In our research, we hypothesize that the use of a more standardized technique was able to limit cases of transient or persistent hypertony.

Chemello et al. have previously reported the double suture technique in BAE implants as an effective surgical variant to lower postoperative IOP spikes. They report a removal rate of the endoluminal sutures in 33% of patients, although no timing protocol for the removal of the sutures was presented. Conversely, in our case series, we removed the thinnest suture at 30 days and the thicker ones at 90 days in all patients following a step-by-step timing of the flow modulation, which helped us to better manage the IOP decrease, and minimize the risk of complications [37].

Selena et al. reported that the early removal of sutures was related to a slight increase in hypotony and hypotony-related complications and was also related to a decreased number of medications used by the patients, highlighting that the flow is essential in the formation of a fibrous capsule, which enables a better long-term filtration [38]. Concerning the hypertensive phase, Chemello et al. speculated that the presence of a double endoluminal suture allows the creation of a space between the sutures that enables a more aqueous flow and results in fewer hypertensive episodes [37]. We also hypothesize that the gradual increase in the flow through the tube and the bleb, which occurs with our postoperative management, is potentially another reason why we did not observe a hypertensive phase. It has been well described that the bleb wall thickening, which is responsible for the hypertensive phase, is associated with copious, unmodulated early flows [36,39]. Breckenridge et al. measured the flow resistance with different endoluminal Prolene sutures in vitro. The results of the study showed that the configuration of a 3-0 Prolene suture in the lumen of the device with venting slits and a 7-0 Vicryl spatulated suture provided the most appropriate flow in the range of physiologic IOPs [31]. The limitation of in vitro studies is that the outflow facility is not evaluated and that correlation with clinical practice remains limited. Different in vitro approaches with various suture sizes showed success rates ranging from 80 to 93.4% [29,40] Marchini et al. used a combination of a 5-0 Prolene endoluminal suture with 7-0 absorbable ligature, a 22 G entry hole, and viscoelastic left in the anterior chamber [40]. In comparison to this study, the use of two endoluminal Prolene sutures allowed us to avoid the use of viscoelastic in the anterior chamber. This is of remarkable interest considering that viscoelastic is known to cause IOP spikes in occluded shunts. Another step-by-step system has been proposed by Sharkawi et al., who showed the results of an occluding stent inserted through the entire length of a silicone tube with or without external ligature. Postoperative outflow was increased through the stepwise removal of the endoluminal suture, which was retracted by 5 mm after at least 6 weeks following surgery and was completely removed after 12 weeks [18]. Even if they reported low rates of HRCs, the abovementioned stepwise technique, as described by the authors, required the use of an operating room to be performed. The advantage of our techniques is that both the thinner and thicker sutures can be easily removed at the slit lamp without the need for an operating room, without opening the anterior chamber, and without the need for specialized instruments.

The main limitation of this research is the limited sample size, which may prevent the findings from being generalizable. Moreover, the lack of a control group is a significant shortfall in this research.

## 5. Conclusions

In conclusion, in our series, none of the patients developed early hypotony-related complications due to the use of a second endoluminal suture. Furthermore, the possibility of a safe and multiple-step regulation of the flow allowed us to manage the hypertensive phase through the removal of the second suture rather than a massive use of IOP-lowering medications. We speculate that better flow modulation can both ensure fewer early postoperative complications and allow the formation of a fibrous capsule more prone to effective long-term filtration. Other prospective studies that use a control group are needed to verify the accurate adoption of this technique and can lead to an improvement in the long-term success of the surgery. Considering its efficacy and the cost-effectiveness of the procedure, the improvement in the safety profile of the BAE implants allows surgeons to expand the indication of non-valved glaucoma implants.

## Figures and Tables

**Table 1:** Summary of gender, age preoperative IOP, and IOP value at each follow-up and if therapy was used.

	Gender	Age	History	IOP Baseline	IOP 1 Day	T	IOP 7 Days	T	IOP 14 Days	T	IOP 30 Days	T	IOP 30 Days after Small Prolene Suture Removal	T	IOP 45 Days	T	IOP 60 Days	T	IOP 90 Days	T	IOP 90 Days after Thick Prolene Suture Removal	T	IOP 6 Months	T	IOP 9 Months	T	IOP 12 Months	T
1	M	71	Uveitic glaucoma	24	16		15		16		18		13		12		14		16		12		12		12		13	
2	F	78	Neovascular glaucoma	26	18	*	16	*	17	*	17	*	15	#	14		17	*	18	*	14	#	15	*	11	*	12	*
3	F	64	POAG, previous ExPRESS	22	20	*	19	*	18	*	18	*	17	#	15		19	*	19	*	13	#	12		13		14	
4	M	69	POAG, previous trabeculectomy	30	21	*	23	**	21	**	22	**	19	#	16		21	*	20	*	15	#	16	*	11	*	10	*
5	F	70	Uveitic glaucoma, previous trabeculectomy	25	16		19		20	*	17	*	11	#	12		13		15		10		14		15		13	
6	F	68	POAG, previous deep sclerectomy	24	11		15		14		16		16		16		15		13		9		13		14		13	
7	M	75	Neovascular glaucoma	28	18		21		17	*	15	*	14	#	13		16		14		11		15		17	*	12	*
8	M	67	Uveitic glaucoma, previous trabeculectomy	27	20	*	14	*	15	*	17	*	13	#	15		13		16		8		13		15		14	
9	M	72	POAG, previous ExPRESS	35	23	**	22	**	18	**	21	**	17	#	14		17		18		13		11		16		15	
10	F	69	POAG	32	9		10		14		15		15		11		19	*	20	*	12	#	15	*	12	*	11	*

M: male; F: female; IOP: intraocular pressure; T: therapy; POAG: primary open-angle glaucoma. * Dorzolamide hydrochloride, timolol maleate preservative-free drops twice daily. ** Acetazolamide tablets (1/2 tablet, 3 times per day + dorzolamide hydrochloride, timolol maleate preservative-free drops twice daily. # Suspended ocular hypotensive therapy.

## Data Availability

The data that support the findings of this study are available from the corresponding author, MMC, upon reasonable request.

## Supplementary Materials

The following supporting information can be downloaded at: https://www.mdpi.com/article/10.3390/vision7010014/s1, Video S1: First Prolene suture intraoperative removal.
